# Exploration of Commonly Used Tests to Assess Physical Qualities in Male, Adolescent Rugby League Players: Discriminative Validity Analyses and Correlations with Match Performance Metrics

**DOI:** 10.3390/sports13070204

**Published:** 2025-06-24

**Authors:** Michael A. Carron, Aaron T. Scanlan, Thomas M. Doering

**Affiliations:** 1 School of Health, Medical and Applied Sciences, Central Queensland University, Rockhampton, QLD 4702, Australia; a.scanlan@cqu.edu.au (A.T.S.); t.doering@cqu.edu.au (T.M.D.); 2 Health, Education, Lifestyle, and Performance (HELP) Laboratory, St Brendan’s College, Yeppoon, QLD 4703, Australia

**Keywords:** development, paediatric, performance, GPS, video analysis, physical activity

## Abstract

Tests assessing physical qualities are regularly used in youth rugby league teams for various functions. However, the utility of such tests is under-explored in this population. In this way, tests are commonly examined in terms of how well they can differentiate performances between groups that are expected to differ and how they relate to outcomes in actual competitive contexts. Therefore, the purpose of this exploratory study was to investigate the discriminative validity and relationships to match performance metrics of frequently used tests to assess physical qualities in male, adolescent rugby league players. Anthropometric (standing height and body mass) and fitness-related (20 m linear sprint, 505-Agility Test, L-run Test, medicine ball throw, countermovement jump, one-repetition maximum back squat, bench press, and prone row tests, and Multistage Fitness Test) physical qualities were measured using common tests in 42 players (16.1 ± 1.3 years). Test outcomes were compared between players in different age and positional groups for discriminative validity analyses. Relationships between test outcomes and match performance metrics gathered via global positioning system and video analysis were also determined. Compared to younger players (14–15 years), older players (16–18 years) had significantly better fitness-related physical qualities (*p* < 0.05, *d* = −1.78–1.66), but similar anthropometric qualities (*p* > 0.05, *d* = −0.45–0.20). Significant, *moderate* correlations (*p* < 0.05, *r* = 0.56–0.70) were found between (1) one-repetition maximum (1-RM) back squat and relative (per min) high-speed running distance and maximum velocity in matches; (2) 20-m sprint time and relative total distance; (3) 505-Agility Test time and relative line breaks; and (4) height and relative unsuccessful tackles. Consequently, commonly used fitness-related tests demonstrate discriminative validity in detecting differences between age groups, with standing height and the 1-RM back squat showing promising utility given their associations with key match metrics in adolescent rugby league players.

## 1. Introduction

Team success during rugby league match-play is predicated on scoring more points via tries, conversions, and field goals than the opposing team. To achieve success in matches, rugby league players repeatedly engage in various physical acts during both offensive and defensive scenarios, including high-intensity runs, precise directional changes, and collisions [1]. In turn, performing these demanding tasks is heavily dependent on possessing well-developed anthropometric and fitness-related physical qualities, such as body mass, linear speed, change-of-direction (COD) speed, muscular power, muscular strength, and aerobic capacity [2]. Indeed, the importance placed on effectively developing these physical qualities has been previously documented among coaches working with adolescent rugby league players [3]. Accordingly, coaching staff of adolescent players in various settings, including schools, utilise periodic anthropometric and fitness testing to assess key physical qualities for various practical functions. For example, coaching staff may implement testing strategies to differentiate physical qualities between players for selection and/or other grouping purposes (i.e., discriminative validity) [4]. Moreover, coaching staff likely want to identify which physical qualities translate most strongly to match performance to assess player potential and readiness for competition [5].

Ensuring a test possesses adequate discriminative validity is essential before it is applied in practice for the purpose of differentiating between players. Specifically, discriminative validity refers to whether test outcomes that are unrelated between separate groups of players are actually not related [5]. Discriminative validity is typically assessed via determining differences in test outcomes between groups of players who are theorised to have varying physical qualities—for instance, differences between players competing at different playing levels or occupying different playing positions [5,6]. Accordingly, within school settings, players competing in different age-based divisions may be expected to possess different physical qualities given older players have likely accrued a higher training age with increased exposure to coaching [7] and may have undergone greater physical development via maturation during adolescence [7,8,9]. In this regard, differentiating between players of different age groups enables end-users to employ age-specific strategies, and identify underperforming players according to their level for appropriate intervention [10]. Importantly, limited research has been conducted on this topic involving adolescent rugby league players, with existing findings suggesting tests assessing physical qualities may hold strong discriminative validity when discerning performances between adult and adolescent age groups, but that this validity may weaken when discriminating between age groups specifically within adolescent players [6,10]. Likewise, players from different positional groups may possess different physical qualities given the varied competitive demands experienced by backs and forwards documented among male, adolescent rugby league players [10], with limited evidence on this topic strictly within this population. Consequently, current evidence on the discriminative validity of tests assessing physical qualities is limited among adolescent rugby league players, with more data needed for a definitive understanding to be established, particularly in schoolboy populations. Furthermore, existing research examining the validity of tests among adolescent players has not accounted for the most frequently adopted tests in the scientific literature, or key physical qualities, such as muscular strength, which has been shown to be important for match performance in male, senior rugby league players [11,12].

Regarding the application of tests assessing physical qualities, it is important to understand how well they relate to real sporting contexts and match performance [5] via correlation analyses between test outcomes and key match performance metrics [13]. In rugby league, global positioning system (GPS) technology and video-based analyses are predominantly used to quantify match performance [14]. However, existing correlation data between test outcomes and match performance are limited to male, senior rugby league players [11,15]. In this previous research, significant (*p* < 0.05) associations were documented between CMJ height and the number of times beating an opposing player (*r* = 0.44) [15], and between 3-RM bench press and back squat loads and tackling-related metrics (*r* = 0.70–0.77) [11]. However, no evidence exists regarding the relationship between commonly used tests to assess physical qualities and match performance metrics among adolescent rugby league players. Such population-specific data is crucial to elucidate, given that the physical qualities [6] and match demands [1] in adolescent rugby league players differ from senior players.

Therefore, the purpose of this exploratory study was to investigate the discriminative validity and relationships with match performance metrics of the most common tests and testing protocols used to measure physical qualities in male, adolescent rugby league players identified in the literature [8]. More precisely, this study sought to determine whether test outcomes: (1) demonstrate discriminative validity between players from different age (14–15 years vs. 16–18 years) and positional groups (backs vs. forwards); and (2) correlate with match performance metrics gathered using GPS and video-based technologies.

## 2. Methods

### 2.1. Subjects

Schoolboy, adolescent rugby league players (*n* = 42, 16.1 ± 1.3 years) were recruited from one secondary school in Australia to participate in this study. Players were eligible to participate if they were free from injury and illness and had completed at least six months of rugby league training (including field training and strength and conditioning) prior to testing. For discriminative validity analyses between age groups, players were eligible to participate if they had competed in the first-grade competition in either the 14–15 years age group (*n* = 19, 14.8 ± 0.4 years) or the 16–18 years age group (*n* = 23, 17.2 ± 0.7 years) across the 2023 schoolboy season. For comparisons between playing positions and correlation analyses, players were eligible to participate if they had competed in the first-grade 16–18 years competition (total sample, *n* = 16, 17.2 ± 0.7 years; backs, *n* = 8, 17.3 ± 0.9 years; forwards, *n* = 8, 17.2 ± 0.6 years) across the 2023 schoolboy season. Written informed consent was obtained from all players and their legal guardians before study commencement. Ethical approval for this study was obtained from the Human Research Ethics Committee (#23570).

### 2.2. Procedures

#### 2.2.1. Study Overview

An observational, exploratory design was adopted to assess the discriminative validity and correlations to match performance metrics of the most common tests and testing protocols used to measure physical qualities in male, adolescent rugby league players identified in the literature [8].

#### 2.2.2. Tests Assessing Physical Qualities

Table 1 outlines all tests and testing protocols used to assess physical qualities in the present study. Players completed three trials of the 20-m linear sprint, 505-Agility Test, L-run Test, MBT, and CMJ, and were permitted 60 s of passive standing rest between each trial, and between each test. For 1-RM tests, players were permitted 180 s of seated rest between each of five attempts. The best outcome from each trial/attempt was recorded for analyses in each test. The MSFT was completed only once. Tests were implemented by the lead researcher, who has demonstrated strong retest reliability in administering these tests in a similar cohort (intraclass correlation coefficient = 0.733–0.999; coefficient of variation = 0.05–4.02%) [16].

#### 2.2.3. Discriminative Validity Assessment

To assess discriminative validity, test outcomes were compared between age groups (14–15 years vs. 16–18 years), and between positional groups (backs vs. forwards) strictly in the 16–18 years age group. The final sample size for each test within each group differed due to varied player availabilities across testing sessions (14–15 years: *n* = 19; 16–18 years: *n* = 19–23; backs: *n* = 7–8; forwards: *n* = 6–8). All tests were administered across two sessions separated by 48 h at the end of a 10-week periodised pre-season phase. Players were instructed to refrain from vigorous activity, consume their typical diet, and undertake their normal daily schedules (i.e., sleep, hydration, nutrition, and school participation) for 48 h prior to each testing session [17]. The first testing session consisted of standing height, body mass, Σ4 skinfold thickness, MBT, CMJ, and 1-RM bench press, back squat, and prone row tests, completed within an air-conditioned (~22 °C), indoor gym facility. The second testing session consisted of the 20-m linear sprint test, 505-Agility Test, L-run Test, and MSFT, completed on an outdoor, regulation (100 m in length and 68 m in width) rugby league field.

**Table 1:** The most common tests and testing protocols identified in the literature [8] and used in this study to assess physical qualities in male, adolescent rugby league players.

Test	Protocol
Standing height	A stadiometer (SECA 213, SECA Corp; Hamburg, Germany) was used; players stood upright with their head positioned in the Frankfurt plane and in bare feet. Standing height was measured to the nearest 0.1 cm.
Body mass	Electronic scales (SECA 813, SECA Corp; Hamburg, Germany) were used; players wore standard rugby league training attire in bare feet. Body mass was measured to the nearest 0.01 kg.
Σ4 skinfold thickness	Measurements taken at biceps, triceps, subscapular, and supra-illiac sites. Harpenden callipers (John Bull, British Indicators Ltd.; London, England) were used to assess skinfold thickness in accordance with The International Society for the Advancement of Kinanthropometry guidelines.
20 m linear sprint	Players stood in a split stance with toes on the leading foot aligned with marking tape 50 cm behind the first timing gate. Single-beam electronic timing gates (SmartSpeed Pro, Fusion Sport; Brisbane, Australia) were used to record 20 m sprint time to the nearest 0.001 s with gates set at 0 and 20 m.
505-Agility Test	Players stood in a split stance with toes on the leading foot aligned with marking tape at 0 m. Players sprinted 15 m forwards before contacting a marked line with their preferred foot and turning 180° back towards the start for a further 5 m. A single-beam electronic timing gate was positioned 10 m from the start to record performance time to the nearest 0.001 s across the 5 m directly before and after the turn.
L-run Test	Players stood in a split stance with toes on the leading foot aligned with marking tape 50 cm behind the timing gate. Players sprinted to a dome marker positioned 5 m in front, before turning 90° in the designated direction and sprinting to another dome marker positioned 5 m away and returning on that same course back to the starting point, while always moving in a forward direction and around the outside of the dome markers. Players completed the first turn at 90°, second turn at 180°, and final turn at 90°. A single-beam electronic timing gate was positioned at 0 m to record performance time to the nearest 0.001 s.
Multistage Fitness Test	The last successful shuttle completed in the MSFT was used to estimate maximal oxygen uptake (mL·kg^−1^·min^−1^) via a valid equation [18]. Players partook in repeated 20 m shuttle runs that progressively increased in pace as indicated by audio cues. Testing concluded when players failed to complete two successive shuttles successfully or stopped voluntarily.
Medicine ball throw	Players threw a 2 kg medicine ball (Celsius; Sydney, Australia) horizontally to achieve the maximum distance (m) possible. Players were seated on a flat bench with their back in contact with a wall, knees flexed to 90°, and feet contacting the floor. A measuring tape was fastened to the floor at the wall below the player when sitting on the bench with distance measured to the nearest 0.1 cm in a direct line to the initial floor marking made by the ball, which was covered in chalk.
Countermovement jump	Players jumped as high as possible with a countermovement phase to displace the highest vane possible on a yardstick device (Swift; Lismore, Australia). A self-selected squat depth and arm swing were permitted during the countermovement with jump height measured to the nearest 1 cm.
One-repetition maximum	One-repetition maximum tests for bench press, back squat, and prone row exercises were conducted, in this order, in accordance with procedures specified by the National Strength and Conditioning Association. All tests were conducted using a 2.13 m Olympic bar (20 kg). The bench press was performed maintaining a supine position on a flat bench with hands pronated using a hook grip. The back squat was performed using a high position and a pronated hook grip, with a self-selected footing, where players were required to squat until their thighs were parallel to the floor. The prone row was performed laying in a prone position on a flat bench, where players pulled the bar towards their body until making contact with the underside of the bench (12 cm thickness) using a pronated hook grip.

#### 2.2.4. Correlations Between Test Outcomes and Match Performance Metrics

For correlation analyses, players completed the same tests across two separate sessions during the in-season phase immediately following a series of competitive matches. More precisely, players completed three consecutive weekly matches in weeks 12–15 of the 16-week in-season. Players then completed testing one week later in the same manner as described for discriminative validity analyses. The final sample size for each test differed due to varied player availabilities across testing sessions (*n* = 11–16). Various metrics were measured via GPS and video-based techniques during each match as performance indicators.

#### 2.2.5. Global Positioning System Procedures and Metrics

Prior to each match, a microsensor device with GPS technology (Catapult S5, OptimEyeTM S5, firmware version 7.22, dimensions: 96 mm × 52 mm × 14 mm; mass: 67 g; Catapult Sports, Melbourne, Australia [which remained firmware version 7.22 for the duration of the study]) was placed securely inside a manufacturer-supplied vest (Catapult Sports; Melbourne, Australia) worn by each player throughout match-play. The same device was worn across all matches by the same player to avoid inter-device variability in outcomes [19]. Each device was turned on at least 20 min before the warm-up prior to matches, which were held on outdoor grassed fields with no obstructions to adequately synchronise with satellites. GPS technology was used to quantify peak velocity (m·s^−1^), total distance covered (m), and total distance covered performing high-speed running (HSR) > 5 m·s^−1^ (m). The cut-point of >5 m·s^−1^ chosen to detect HSR has been previously established across the literature in rugby league players [20]. Both total distance and total HSR distance were determined relative (per min) to the total individualised time each player was on the field accumulated across all matches. Total time on the field excluded half-time breaks and interchanged periods when not actively participating in matches, which were manually recorded by the lead researcher. Across the three matches combined, players had an average total time of 136.7 ± 41.5 min (range: 59.8–188.5 min) on the field.

#### 2.2.6. Video-Based Procedures and Metrics

Video analyses were used to measure various match performance metrics adapted from an established rugby league video analysis framework [21]. Specifically, modifications to the original framework included development of attacking metrics that mirrored the descriptors provided for defensive metrics. For instance, descriptors provided in the original framework for a dominant tackle position on defence were modified for application to line contacts on offence. In this way, players undergoing line contacts were scored based on the position (e.g., positive) they occupied following line contact when in possession of the ball. While several metrics were provided in the original framework, the limited counts observed for some performance metrics (e.g., fends) precluded their inclusion, so only metrics that were performed at least once in a match by all players were included for analysis. Consequently, six performance metrics (Table 2) derived from video footage were included in this study. Video footage was recorded for each match by a professional videographer using a Sony HDC-4300 camcorder (60 frames per second at 1/120th shutter). Video footage was analysed and coded by the lead researcher using Hudl (Agile Sports Technologies Inc., Lincoln, NE, USA). Video-based performance metrics were recorded as frequency counts tabulated across all matches and made relative (per min) to total individualised time on the field for each player. All video-based performance metrics were analysed by the lead researcher, with 101 video clips (21 min and 53 s of footage) randomly analysed on a separate occasion to determine the intra-rater reliability across metrics (*r* = 0.823, Cohen’s Kappa = 0.905).

### 2.3. Statistical Analysis

Normality and homogeneity of variance for each test outcome variable were confirmed using the Shapiro–Wilk and Levene’s tests. Accordingly, all data are reported descriptively as mean ± standard deviation (SD). For discriminative validity analyses, separate independent *t*-tests were used to detect significant differences in test outcomes between age groups and between positional groups. Magnitudes of differences for pairwise comparisons were determined as mean differences (with 95% confidence intervals [CI]) and Cohen’s *d* effect size calculated using a published spreadsheet [22] (with 95% CI) with effect magnitudes interpreted as *trivial*, <0.20; *small*, 0.20–0.49; *medium*, 0.50–0.79; or *large*, ≥ 0.80 [23]. Pearson correlations (r) were also determined between test outcomes and match performance metrics. Correlation magnitudes were interpreted as *trivial* = <0.20, *small* = 0.20–0.49, *moderate* = 0.50–0.79, or *large* = ≥0.80 [23]. Statistical significance was accepted where alpha was <0.05. All analyses were conducted using IBM SPSS software (version 24 IBM Corp.; Armonk, NY, USA) or Microsoft Excel (version 15, Microsoft Corp.; Redmond, WA, USA).

## 3. Results

### 3.1. Discriminative Validity Analyses

Table 3 shows the descriptive data for test outcomes alongside discriminative validity analyses between age groups. The 16–18 years group performed significantly better in all fitness-related tests (*p* ≤ 0.05, *d* = −1.78–1.66, *medium*–*large*), but not in anthropometric tests (*p* > 0.05, *d* = −0.45–0.20, *trivial*–*small*). Table 4 shows the descriptive data for test outcomes with discriminative validity analyses between positional groups. No significant differences (*p* > 0.05, *d* = −0.40–0.73, *trivial*–*large*) were evident for any test outcome between positional groups.

### 3.2. Correlation Analyses

Table 5 shows the correlation coefficients between anthropometric qualities and match performance metrics. A significant, *moderate* correlation was found between standing height and unsuccessful tackles (*p* < 0.05, *r* = 0.57).

Table 6 shows the correlation coefficients between fitness-related physical qualities and match performance metrics. Significant, moderate correlations were found between 1-RM back squat performance and relative HSR distance as well as peak velocity during matches (*p* < 0.05, r = 0.56–0.70). In addition, significant, moderate correlations were apparent between 20-m linear sprint time and relative total distance (r = 0.61), between 505-Agility Test time and relative number of line breaks (r = 0.60), and between standing height and relative number of times accelerating into tackles (r = 0.57).

## 4. Discussion

This exploratory study is the first to assess the discriminative validity of the most common tests and testing protocols used to measure physical qualities in male, adolescent rugby league players. Moreover, it is the first study to examine the correlations between physical qualities and match performance metrics derived from both GPS and video-based technologies in wider rugby league research. The main findings from this study are as follows: (1) fitness-related but not anthropometric tests differentiate physical qualities between 14–15 years and 16–18 years first-grade schoolboy, adolescent players; (2) similar physical qualities were apparent between backs and forwards in 16–18-year-old players using these tests; and (3) outcomes from standing height, 20-m sprint, 505-Agility Test, and 1-RM back squat assessments were significantly correlated with match performance metrics among schoolboy, adolescent rugby league players.

Findings from this study suggest that the most common tests and testing protocols used to measure fitness-related physical qualities in male, adolescent rugby league players identified in the literature [8] can be used to differentiate between age groups within a first-grade school context. In this way, most tests may hold suitable discriminative validity to apply for player selection purposes perhaps when factoring in various sources of evidence to decide on team line-ups or identifying potential players with suitable qualities. Moreover, given that these tests can differentiate between age groups within the school context, they may be used to assign players to appropriate playing levels. In this way, participating within an appropriate competition relative to their physical ability may increase player enjoyment, improve long-term retention in sports participation, and ultimately strengthen health outcomes in this population. Importantly, results from this study partially agree with those reported previously by Dobbin et al. [6], who found that the CMJ, MBT, and prone Yo-YoIRT differentiated (*d* = 0.23–1.00) between male youth (15.1 ± 0.8 years) and academy (17.5 ± 2.0 years) adolescent rugby league players, with superior results among the older age group. However, these authors [6] reported that 20-m linear sprint (*d* = −0.06) and COD speed (*d* = 0.10) tests did not differentiate between youth and academy players, but height and body mass were greater among academy players. Indeed, these contrasting results reported by Dobbin et al. [6] may be interrelated given that they speculated that the heavier mass among academy players may require greater relative force generation to overcome body inertia during the accelerative phases of the 20-m sprint and COD tests. Stemming from this supposition, variations in findings between studies may relate to the nature of the samples investigated, whereby players in the older age group in the study by Dobbin et al. [6] were heavier than the players in the present study of a similar age (87.5 ± 11.7 kg vs. 78.2 ± 9.1 kg), potentially explaining the discrepancies reported between age groups for linear and COD speed. Additionally, the present findings regarding better linear and COD speed among older players may be explained by the introduction of specialised resistance training among this group and not younger players in the school training environment examined. While the training regimes of the different age groups were not specified in previous research by Dobbin et al. [6], the introduction of specialised resistance training among older players in the present study likely enhanced their ability to rapidly generate force and overcome body inertia while improving technical capacities in linear sprinting and COD manoeuvres [24]. Importantly, differences in performance outcomes in the present study may stem from the older group (16–18 years) having undergone greater maturation than the younger group (14–15 years). Indeed, previous research has shown that adolescent rugby league players (in under-13, -14, and -15-year categories) with greater maturity demonstrate superior physical qualities than their less matured counterparts [7]. However, the present study is limited in this way, given maturation status was not assessed due to precluding factors prohibiting its measurement. Nonetheless, the findings from the present study are broadly consistent with the current body of evidence concerning expected differences in anthropometric and physical qualities among adolescents [6,25].

The findings from the current study show non-significant differences in test outcomes between positional groups within first-grade, 16–18 years players. However, it is worth noting that body mass, Σ4 skinfolds, and 1-RM bench press, back squat, and prone row performances were *moderately* (*d* = 0.54–0.73) greater in forwards compared to backs. Moreover, non-significant differences may be a reflection of the small sample in this analysis. As such, contrary to findings from the present study, Gabbett [10] found that male, adolescent rugby league players (16–18 years) in the prop position had significantly greater (*p* < 0.05) height, body mass, and Σ4 skinfold thickness, and significantly worse (*p* < 0.05) 10- and 20-m linear sprint times, COD performance time, and aerobic capacity than all other positions (i.e., hookers, halves, backrowers, and outside backs). However, Gabbett [10] examined sub-elite rugby league players who likely occupy certain positions more consistently throughout a season compared to players in school settings who may rotate between positions due to more generalised roles. As such, the similarities in physical qualities observed between backs and forwards in the present study may be explained by homogenous training strategies in school contexts which focus on general physical development across positional groups due to resource and time constraints among student-athletes [26]. Moreover, playing positions were categorised more broadly as backs and forwards in the present study compared to the more precise positional groups adopted by Gabbett [10], given that this approach has been used elsewhere [27] and maximises the sample size for each positional group due to the restricted recruitment within a single school in this study. In this way, broader positional grouping may diminish the extent of potential position-related differences in physical qualities.

This is the first study to examine correlations between tests assessing physical qualities and match performance metrics among schoolboy, adolescent rugby league players. Although most correlations were non-significant and *trivial*-to-*small* in magnitude, some notable relationships were observed. Specifically, correlation analyses revealed that players with greater maximal lower-body muscular strength, assessed via the 1-RM back squat test, tended to cover greater relative HSR distance and achieve greater peak velocities during matches. Although no studies have examined correlations between performance in the 1-RM back squat test and match performance metrics among schoolboy, adolescent rugby league players, Johnston et al. [28] showed that male, sub-elite rugby league players (19.2 ± 0.7 years) categorised as having a high 3-RM back squat performance covered greater HSR distances (>5.1 m·s^−1^) during matches than those categorised as having a low 3-RM back squat performance (*d* = 1.00, *p* = 0.01). Consequently, the collective findings across previous research [28] and from this study indicate that maximal lower-body muscular strength may be a critical correlate of intense running outputs in rugby league match-play among adolescent players. These findings might be reflective of the various adaptive mechanisms associated with resistance training to develop strength-related qualities such as enhanced musculotendinous stiffness, motor unit recruitment, and rate of force development [24]. Moreover, a significant, positive correlation was found between standing height and unsuccessful tackles. In this regard, the players investigated in the current study were likely undergoing varied rates of physical growth as commonly seen in the adolescent developmental phase [29]. Given that heightened rates of physical growth are associated with motor incoordination [29], players who were taller may have exhibited poorer movement execution and subsequently been more likely to miss tackles.

Regarding other notable relationships, a significant, positive correlation was found between 20-m sprint time and relative total distance, indicating that slower players cover greater distances per minute of play. Indeed, this finding suggests that the ability to maintain movement consistently across matches (i.e., high average speed) is not predicated on the ability to simply sprint quickly in a single bout, with previous research in male, professional rugby league players also suggesting other physical attributes (repeated-sprint ability, repeated-high-intensity running capacity, and aerobic capacity) have limited association with relative total distance in matches [30]. Consequently, a combination of physical qualities alongside other factors (e.g., technical and tactical elements) likely contributes to the relative total distance covered in rugby league match-play, which warrants further exploration. Similar to this finding, a significant, positive correlation was also found between 505-Agility Test time and relative line breaks, indicating that players with slower COD speed penetrate the defensive line more often. Indeed, this correlation indicates that executing line breaks may not overly depend on COD speed, which is partially supported by data reported during rugby union match-play demonstrating that most line breaks involve no change in running angle [31]. Alternatively, this result might relate to the design of the 505-Agility Test, whereby players are required to perform a pre-planned 180° turn that more resembles gaining position within the defensive line between tackles during matches. In contrast, a test involving a COD while maintaining movement in a forward direction (e.g., side-step manoeuvre) [32] and involving a reactive component (e.g., simulated opponent) [33] may better translate to player potential in breaking the defensive line during matches via responding to and evading opponents. As further reasoning, the correlations between 20-m sprint time and relative total distance, and between 505-Agility Test time and relative line breaks may have been impacted by the narrow dispersion in performance times observed in these tests (e.g., 20 m linear sprint: 3.094 ± 0.203 s, *n* = 42; 505-Agility Test: 2.282 ± 0.070 s, *n* = 41). This tight range in performance times across the sample in this study may have been due to similarly developed accelerative abilities among players in this population [34] and in turn could have influenced the correlations observed [35].

This study is not without limitations, which should be acknowledged when interpreting the findings. First, the correlation analysis involved a single schoolboy team with some players not able to complete all tests, limiting the final sample sizes. Small samples increase the likelihood of encountering statistical testing errors in correlation analyses, [36] which may have also contributed to the counterintuitive relationships observed between some variables. Moreover, many physical (and non-physical) aspects likely underpin the complex multifaceted nature of competitive performance in team sports [35,37], with their associations not necessarily being linear in shape. However, the small sample limited the number of datapoints that could have been used to uncover more complex relationships via multivariate and non-linear analyses in this study. Therefore, future work is encouraged to expand on the analyses conducted in this work by recruiting larger samples from multiple schools. Ultimately, interpretations of the correlational findings from this exploratory study should be carried out with caution—especially those that are counterintuitive—given that they are population-specific and limited to the small sample able to be recruited. Second, the sample comprised two different age groups both competing at a first-grade level, as opposed to different levels or grades within the same age group. However, given that players compete within their respective chronological age group in school settings, this approach was logical for the current study. Third, a selection of tests and testing protocols identified as the most commonly used to measure physical qualities in male, adolescent rugby league players in the literature [8] was utilised in this study. Consequently, contemporary tests gaining increased popularity in recent years such as the isometric midthigh pull [38], and tests implemented by industry bodies in practice but not the literature, were not included in this study. Future research should examine other tests in male, adolescent rugby league players as they become more widely adopted in research and practice. Lastly, the video analysis was limited to six performance metrics due to the infrequent occurrence of the wider set of metrics during matches across the players investigated. This approach restricted the scope of performance analysis able to be performed from the original video analysis framework [21], and may have limited our ability to elucidate meaningful relationships between physical qualities and match performance.

### Practical Applications

The present findings show that common tests used to measure fitness-related physical qualities in male, adolescent rugby league players appear to adequately differentiate between first-grade age groups (i.e., 14–15 and 16–18 years) in an Australian schoolboy context, meaning they may be useful for player assignment to appropriate playing levels, which, in turn, may enhance long-term retention and health outcomes in this population. The lack of differences in physical qualities between positional groups may be due to player development strategies within school settings being generic in nature rather than specific to playing position due to time and resource constraints. Consequently, it is probable that physical qualities may be rather homogenous across positional groups when players are categorised broadly as backs and forwards in a schoolboy, adolescent cohort. Furthermore, while findings from the present study show that most physical qualities did not significantly correlate with match performance metrics, some notable relationships were observed. In this way, the significant associations between 1-RM back squat performance and relative HSR distance as well as peak velocity during matches indicates that this test may provide outcomes that inform player potential to execute intense running demands in match-play. In turn, the significant correlation between standing height and unsuccessful tackles might inform player selection strategies for specific positions (e.g., those that require extensive tackling requirements) or encourage suitable technical intervention to circumnavigate this issue in taller players.

## 5. Conclusions

The most common tests and testing protocols used to measure fitness-related, but not anthropometric qualities, in male, adolescent rugby league players significantly differentiated between age groups in first-grade schoolboy players. Moreover, there were no significant differences in physical qualities between playing positions when generalised as backs and forwards, likely due to homogenous development strategies being applied across positional groups in school settings. In turn, while most correlations between physical qualities and match performance metrics were non-significant and *trivial*-to-*small* in magnitude, the 1-RM back squat test and standing height may hold some translation to match-play given their significant, *moderate* associations with performance metrics. Future studies involving larger samples of adolescent rugby league players across diverse school settings are encouraged to build upon these findings, contributing to a broader and more robust evidence base on this topic. Given the exploratory nature of the present study, the findings may help guide the generation of hypotheses in future confirmatory research with adequate error control [39]. In this regard, based on the present findings, future studies may hypothesise that commonly used tests assessing fitness-related physical qualities effectively discriminate between high-level (e.g., first-grade), adolescent, schoolboy rugby league players participating in different competitions predicated on chronological age (e.g., 14–15 years vs. 16–18 years). Moreover, it may be hypothesised that lower tackling proficiency is related with a taller standing height and that stronger 1-RM back squat performance is related with greater high-intensity running outputs in matches among players.

## Figures and Tables

**Table 2:** Video-based match performance metrics used in this study that were taken, or adapted, from an established rugby league video analysis framework [21].

Metric	Description
Defensive	
Accelerates into line for tackles	The tackler deliberately speeds up towards the line before making contact.
Unsuccessful tackles	The ball carrier was not held by the tackler; the ball or a ball-carrier’s hand did not make contact with the ground or was not stopped from making further progress towards the try line.
Dominant tackles	The ball carrier was held by the tackler, and advanced backwards (negative progress) towards their own try line.
Offensive	
Dominant line contact	The ball carrier had gained territory towards the opposing try line during the contact phase.
Accelerates into line contact	The ball carrier deliberately speeds up towards the line before making contact.
Line break	The ball carrier passes what is determined as the defensive line and advances toward the opposition try line.

**Table 3:** Descriptive data and discriminative validity analyses of common tests used to assess physical qualities in male, adolescent rugby league players via comparisons between age groups.

Test	Age Group		*p*	Mean Difference (95% CI)	Cohen’s *d* (95% CI)
	14–15 Years	16–18 Years			
Standing height (cm)	178.5 ± 4.9	179.4 ± 4.3	0.566	−0.85 (−3.80, 2.11)	0.20 (−0.43, 0.82)
Body mass (kg)	79.2 ± 10.5	78.2 ± 9.1	0.738	1.06 (−5.32, 7.45)	−0.10 (−0.73, 0.53)
Σ4 skinfolds (mm)	52.77 ± 9.46	48.47 ± 9.85	0.174	4.31 (−1.99, 10.60)	−0.45 (−1.08, 0.19)
20 m linear sprint (s)	3.125 ± 0.138	2.991 ± 0.137	0.003 *	0.13 (0.05, 0.22)	−0.98 (−1.62, −0.33)
505-Agility Test (s)	2.232 ± 0.152	2.331 ± 0.089	0.013 *	−0.10 (−0.18, −0.02)	0.81 (0.17, 1.45)
L-run test (s)	5.995 ± 0.268	5.585 ± 0.191	<0.001 *	0.41 (0.27, 0.56)	−1.78 (−2.51, −1.06)
MSFT (mL·kg^−1^·min^−1^)	41.1 ± 5.3	45.3 ± 3.9	0.006 *	−4.21 (−7.14, −1.29)	0.91 (0.27, 1.56)
CMJ (cm)	53.06 ± 8.9	63.2 ± 5.7	<0.001 *	−10.12 (−14.74, −5.49)	1.38 (0.70, 2.06)
MBT (m)	6.79 ± 0.60	7.14 ± 0.54	0.049 *	−0.36 (−0.72, 0.00)	0.62 (−0.01, 1.24)
1-RM bench press (kg)	82.1 ± 13.9	100.5 ± 19.1	0.001 *	−18.33 (−28.94, −7.72)	1.09 (0.44, 1.75)
1-RM back squat (kg)	94.2 ± 17.1	125.7 ± 20.3	<0.001 *	−31.44 (−43.30, −19.58)	1.66 (0.96, 2.30)
1-RM prone pow (kg)	63.2 ± 12.2	82.2 ± 13.3	<0.001 *	−19.02 (−27.04, −11.00)	1.48 (0.80, 2.17)

Notes: Sample size for each test ranged between 39 and 42; data are presented as mean ± standard deviation; * denotes significant difference between groups. Abbreviations: CI, confidence intervals; MSFT, Multistage Fitness Test; MBT, medicine ball throw; CMJ, countermovement jump; 1-RM, one-repetition maximum.

**Table 4:** Descriptive data and discriminative validity analyses of common tests used to assess physical qualities in male, adolescent rugby league players via comparisons between positional groups.

Test	Positional Group		*p*	Mean Difference (95% CI)	Cohen’s *d* (95% CI)
	Backs	Forwards			
Standing height (cm)	182.9 ± 3.0	183.4 ± 9.0	0.901	−0.43 (−7.78, 6.92)	0.08 (−0.97, 1.12)
Body mass (kg)	77.6 ± 7.3	85.3 ± 19.0	0.310	−7.692(−23.52, 8.13)	0.54 (−0.53, 1.60)
Σ4 skinfolds (mm)	46.82 ± 9.69	57.68 ± 17.86	0.168	−10.86 (−26.98, 5.26)	0.70 (−0.33, 1.84)
20 m linear sprint (s)	3.107 ± 0.237	3.080 ± 0.175	0.821	0.03 (−0.23, 0.29)	−0.13 (−1.22, 0.96)
505-Agility Test (s)	2.268 ± 0.159	2.227 ± 0.179	0.688	0.04 (−0.18, 0.26)	−0.24 (−1.38, 0.89)
L-run test (s)	5.691 ± 0.239	5.766 ± 0.337	0.658	−0.08 (−0.44, 0.29)	0.26 (−0.88, 1.39)
MSFT (mL·kg^−1^·min^−1^)	47.9 ± 2.7	47.2 ± 2.9	0.698	0.67 (−3.12, 4.46)	−0.25 (−1.44, 0.94)
CMJ (cm)	63.88 ± 5.72	60.88 ± 9.09	0.443	3.00 (−5.15, 11.15)	−0.40 (−1.39, 0.59)
MBT (m)	6.53 ± 0.59	6.33 ± 0.99	0.640	0.20 (−0.70, 1.09)	−0.24 (−1.26, 0.78)
1-RM bench press (kg)	85.6 ± 4.2	94.3 ± 17.4	0.194	−8.66 (−22.33, 5.01)	0.67 (−0.38, 1.71)
1-RM back squat (kg)	118.8 ± 10.9	130.0 ± 20.0	0.200	−11.25 (−29.34, 6.84)	0.70 (−0.31, 1.71)
1-RM prone row (kg)	75.0 ± 9.3	83.8 ± 14.3	0.169	−8.75 (−21.69, 4.19)	0.73 (−0.28, 1.74)

Notes: Sample size for each test ranged between 11 and 16; data are presented as mean ± standard deviation. Abbreviations: CI, confidence intervals; MSFT, Multistage Fitness Test; MBT, medicine ball throw; CMJ, countermovement jump; 1-RM, one-repetition maximum.

**Table 5:** Pearson correlations (with 95% confidence intervals) between common tests used to assess anthropometric qualities and match performance metrics in male, adolescent rugby league players.

Test	Match Performance Metric								
	Distance	HSR Distance	Peak Velocity	Accelerates into Tackles	Unsuccessful Tackles	Dominant Tackles	Dominant Line Contacts	Accelerations into Line	Line Breaks
Standing height	0.32 (−0.25, 0.73)	0.15 (−0.41, 0.63)	0.09 (−0.46, 0.60)	−0.01 (−0.54, 0.53)	0.57 (0.05, 0.84) *	−0.14 (−0.63, 0.42)	0.19 (−0.38, 0.65)	0.06 (−0.49, 0.57)	−0.02 (−0.54, 0.52)
Body mass	0.24 (−0.31, 0.67)	−0.07 (−0.56, 0.46)	0.00 (−0.51, 0.51)	0.21 (−0.34, 0.66)	0.32 (−0.23, 0.72)	−0.01 (−0.61, 0.49)	0.24 (−0.32, 0.67)	0.22 (−0.33, 0.66)	0.13 (−0.41, 0.60)
Σ4 skinfolds	−0.00 (−0.55, 0.55)	−0.54 (−0.84, 0.01)	−0.24 (−0.70, 0.36)	0.09 (−0.49, 0.61)	0.08 (−0.49, 0.61)	0.29 (−0.31, 0.73)	−0.19 (−0.67, 0.41)	−0.19 (−0.67, 0.41)	−0.06 (−0.59, 0.51)

Notes: * denotes significant correlation (*p* < 0.05). Unshaded cells indicate *trivial* relationships (*r* = <0.20), lightly shaded cells indicate *small* relationships (*r* = 0.20 –0.49), and darker shaded cells indicate *moderate* relationships (*r* = 0.50–0.79). All performance metrics were determined relative to on-field time, except for peak velocity. Abbreviations: HSR, high-speed running.

**Table 6:** Pearson correlations (with 95% confidence intervals) between common tests used to assess fitness-related physical qualities and match performance metrics in male, adolescent rugby league players.

Test	Match Performance Metric								
	Distance	HSR Distance	Peak Velocity	Accelerates into Tackles	Unsuccessful tackles	Dominant Tackles	Dominant Line Contacts	Accelerations into Line	Line Breaks
20 m sprint	0.61 (0.09, 0.87) *	−0.30 (−0.73, 0.31)	−0.23 (−0.69, 0.37)	−0.12 (−0.63, 0.47)	0.36 (−0.24, 0.76)	−0.15 (−0.65, 0.43)	0.04 (−0.52, 0.58)	−0.13 (−0.64, 0.45)	0.20 (−0.40, 0.68)
505-Agility Test	0.32 (−0.32, 0.75)	0.38 (−0.25, 0.78)	0.27 (−0.36, 0.73)	−0.27 (−0.73, 0.36)	0.14 (−0.48, 0.66)	−0.22 (−0.71, 0.41)	0.49 (−0.11, 0.83)	0.51 (−0.10, 0.84)	0.60 (0.04, 0.87) *
L-run test	0.14 (−0.47, 0.66)	0.11 (−0.49, 0.65)	0.15 (−0.47, 0.67)	0.03 (−0.55, 0.60)	0.17 (−0.45, 0.68)	−0.22 (−0.71, 0.40)	0.44 (−0.18, 0.81)	0.45 (−0.17, 0.81)	−0.09 (−0.63, 0.51)
MSFT	−0.30 (−0.76, 0.37)	0.14 (−0.50, 0.68)	0.02 (−0.58, 0.62)	0.48 (−0.17, 0.84)	0.01 (−0.60, 0.61)	0.07 (−0.56, 0.64)	−0.13 (−0.68, 0.51)	−0.15 (−0.69, 0.49)	−0.21 (−0.72, 0.45)
CMJ	−0.09 (−0.55, 0.42)	0.24 (−0.29, 0.66)	−0.01 (−0.50, 0.49)	−0.39 (−0.74, 0.13)	−0.03 (−0.52, 0.47)	−0.48 (−0.79, 0.02)	−0.10 (−0.57, 0.42)	−0.08 (−0.55, 0.44)	−0.06 (−0.54, 0.45)
MBT	−0.02 (−0.53, 0.50)	0.30 (−0.26, 0.70)	0.30 (−0.25, 0.70)	−0.09 (−0.57, 0.45)	−0.13 (−0.60, 0.41)	−0.32 (−0.72, 0.23)	0.38 (−0.17, 0.75)	0.40 (−0.14, 0.76)	0.30 (−0.25, 0.70)
1-RM bench press	0.38 (−0.16, 0.75)	0.01 (−0.46, 0.57)	0.07 (−0.46, 0.57)	0.26 (−0.29, 0.68)	0.30 (−0.25, 0.70)	−0.21 (−0.65, 0.34)	0.33 (−0.22, 0.72)	0.26 (−0.29, 0.68)	0.06 (−0.47, 0.56)
1-RM back squat	0.06 (−0.49, 0.57)	0.56 (0.04, 0.84) *	0.70 (−0.27, 0.90) *	−0.10 (−0.60, 0.45)	0.11 (−0.44, 0.61)	−0.30 (−0.72, 0.28)	0.09 (−0.46, 0.59)	0.10 (−0.46, 0.60)	−0.11 (−0.60, 0.45)
1-RM prone row	0.03 (−0.48, 0.51)	−0.12 (−0.62, 0.35)	0.23 (−0.30, 0.65)	0.18 (−0.35, 0.62)	−0.02 (−0.51, 0.48)	−0.09 (−0.56, 0.43)	0.33 (−0.20, 0.71)	0.31 (−0.22, 0.70)	0.36 (−0.17, 0.72)

Notes: * denotes significant correlation (*p* <0.05). Unshaded cells indicate *trivial* relationships (*r* = <0.20), lightly shaded cells indicate *small* relationships (*r* = 0.20–0.49), and darker shaded cells indicate *moderate* relationships (*r* = 0.50–0.79). All performance metrics were determined relative to on-field time, except for peak velocity. Abbreviations: HSR, high-speed running; MSFT, Multistage Fitness Test; CMJ, countermovement jump; MBT, medicine ball throw; 1-RM, one-repetition maximum.

## Data Availability

The original contributions presented in this study are included in the article. Further inquiries can be directed to the corresponding author.
